# Investigating Menstruation and Adverse Pregnancy Outcomes: Oxymoron or New Frontier? A Narrative Review

**DOI:** 10.3390/jcm13154430

**Published:** 2024-07-29

**Authors:** Kirstin Tindal, Fiona L. Cousins, Stacey J. Ellery, Kirsten R. Palmer, Adrienne Gordon, Caitlin E. Filby, Caroline E. Gargett, Beverley Vollenhoven, Miranda L. Davies-Tuck

**Affiliations:** 1 The Ritchie Centre, Hudson Institute of Medical Research, Clayton, VIC 3168, Australiacaroline.gargett@monash.edu (C.E.G.); miranda.davies@hudson.org.au (M.L.D.-T.); 2 Department of Obstetrics and Gynaecology, Monash University, Clayton, VIC 3168, Australia; kirsten.palmer@monash.edu (K.R.P.); beverley.vollenhoven@monash.edu (B.V.); 3 NHMRC Centre for Research Excellence (CRE) in Stillbirth, Brisbane, QLD 4101, Australia; adrienne.gordon@sydney.edu.au; 4 Women’s and Newborn Program, Monash Health, Clayton, VIC 3168, Australia; 5 Central Clinical School, Faculty of Medicine and Health, University of Sydney, Sydney, NSW 2006, Australia

**Keywords:** menstruation, endometrium, pregnancy, menstrual fluid, adverse pregnancy outcomes, stillbirth, placenta

## Abstract

Not discounting the important foetal or placental contribution, the endometrium is a key determinant of pregnancy outcomes. Given the inherently linked processes of menstruation, pregnancy and parturition with the endometrium, further understanding of menstruation will help to elucidate the maternal contribution to pregnancy. Endometrial health can be assessed via menstrual history and menstrual fluid, a cyclically shed, easily and non-invasively accessible biological sample that represents the distinct, heterogeneous composition of the endometrial environment. Menstrual fluid has been applied to the study of endometriosis, unexplained infertility and early pregnancy loss; however, it is yet to be examined regarding adverse pregnancy outcomes. These adverse outcomes, including preeclampsia, foetal growth restriction (FGR), spontaneous preterm birth and perinatal death (stillbirth and neonatal death), lay on a spectrum of severity and are often attributed to placental dysfunction. The source of this placental dysfunction is largely unknown and may be due to underlying endometrial abnormalities or endometrial interactions during placentation. We present existing evidence for the endometrial contribution to adverse pregnancy outcomes and propose that a more comprehensive understanding of menstruation can provide insight into the endometrial environment, offering great potential value as a diagnostic tool to assess pregnancy risk. As yet, this concept has hardly been explored.

## 1. Introduction

The death of a baby before its life even begins is a devasting outcome of pregnancy. Globally, more than 4 million babies die in the perinatal period (as a stillbirth or neonatal death (NND)) every year [1,2]. In high-income countries, current approaches to reduce perinatal death focus predominantly on care at the end of pregnancy. While gains are being made in reducing late gestation stillbirth [3,4], 85% of perinatal deaths occur in the preterm period (<37 weeks’ completed gestation), with the most common classified causes (excluding congenital anomalies) being spontaneous preterm birth (e.g., preterm premature rupture of membranes (pPROM); 11.5% stillbirths and 38% NND), foetal growth restriction (FGR; 9% stillbirths, where the baby fails to reach its growth potential), or unexplained antepartum deaths, with no cause found (14% stillbirths) [5]. Preeclampsia, a hypertensive disorder of pregnancy, is also a leading cause of maternal morbidity and mortality worldwide [6]. The underlying biological pathways that lead to this continuum of adverse pregnancy outcomes, including preeclampsia and FGR, spontaneous preterm birth and perinatal death, are not fully understood. Even though these outcomes manifest in different ways, ‘placental insufficiency’ is commonly attributed as the driver of adverse outcomes. The causes of poor placental function, however, remain largely unknown.

The evidence demonstrates that the endometrial environment into which the embryo implants is crucial in the establishment and progression of pregnancy, including placental development [7,8,9,10]. Defective decidualisation of the endometrium has been linked with recurrent pregnancy loss, spontaneous preterm birth and preeclampsia [11,12,13,14]. Abnormal menstruation, such as irregular periods or early onset of menarche, has also been associated with the development of preeclampsia and FGR [15] and preterm birth [16]. Despite these correlations, the role of the endometrium and menstruation in contributing to adverse pregnancy outcomes has been entirely understudied. The lack of research in this space to date may be attributed to the difficulty in performing non-invasive assessments of the endometrial environment. With the advent of menstrual fluid research, however, it is now possible to non-invasively collect and measure the components of menstrual fluid that reflect the endometrium around the time of embryo implantation [17,18,19], thus providing a window into this dynamic environment.

**Figure 1:**
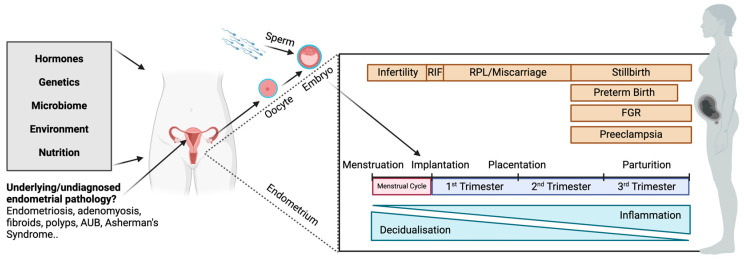
The potential contribution of factors that contribute to the resultant spectrum of adverse pregnancy outcomes. AUB: abnormal uterine bleeding; RIF: recurrent implantation failure; RPL: recurrent pregnancy loss; FGR: foetal growth restriction.

In this review, we reiterate that the endometrial environment into which the embryo implants is an integral component in establishing the trajectory of pregnancy. Abnormal placentation likely stems from impaired decidualisation and inflammation, leading to reduced endometrial receptivity and, therefore, aberrant implantation [20,21]. Just as these are thought to be factors leading to infertility and recurrent pregnancy loss, we emphasise that they can lead to a spectrum of adverse outcomes throughout pregnancy (Figure 1). We highlight several studies of placental insufficiency biomarkers detected in peripheral blood or placental tissues, which are not necessarily representative of the endometrial environment, making it difficult to draw inferences from these associations. We also provide detailed evidence from endometrial biopsy and in vitro studies, including differences in cellular and protein pathways and gene expression, demonstrating the contribution of the endometrium to adverse pregnancy outcomes.

Studying menstruation and menstrual fluid has unrealised potential to better understand the origins of poor placental function. Specifically, there is the prospect for menstrual fluid biomarker screening to be used during preconception care or after adverse events have occurred to guide future pregnancy care. There is also an opportunity to inform the development or repurposing of therapeutics to target specific cellular pathways that underpin poor pregnancy outcomes. Whilst it might seem contradictory to use menstruation, a biological process that signals the very absence of pregnancy, to research obstetric complications and adverse pregnancy outcomes, it provides the perfect window into the endometrial environment of the uterus prior to implantation.

## 2. The Endometrial Environment

### 2.1. Endometrial Composition Changes over the Menstrual Cycle

The endometrium is a heterogeneous tissue comprising epithelial, stromal, stem/progenitor and immune cells. Throughout the menstrual cycle and during pregnancy, the composition of the endometrium changes dynamically in response to circulating ovarian hormones. During menstruation, the functional layer of endometrial tissue sheds, and the endometrial ‘wound’ begins to repair concurrently [22,23]. Following repair and under the influence of rising oestrogen levels during the proliferative phase, endometrial regeneration is likely mediated by stem/progenitor cells to regenerate the glands and vascularised stroma of the functional layer of the endometrium. Regeneration involves the endometrium more than doubling in thickness, growing up to 1 cm of mucosal tissue [23]. After ovulation, the secretory phase involves the differentiation of glandular epithelium into secretory cells that produce mucin-rich secretions for nourishing a future embryo; and stromal cells, which undergo distinct differentiation to become highly secretory epithelioid cells in preparation for embryo implantation. Endometrial glandular epithelium and stroma differentially express proteins throughout the varying phases of the menstrual cycle, and endometrial receptivity is influenced by a suite of molecules [24,25].

The proportion of immune cells in the endometrium also fluctuates dramatically throughout the phases of the menstrual cycle. Previous research shows that the proportion of CD45+ endometrial leukocytes doubles between the late proliferative and late secretory phases and represents up to 90% of all endometrial cells towards the end of the menstrual cycle [17,26]. Of note is the influx of uterine natural killer (uNK) cells, neutrophils and monocytes, which differentiate into macrophages, particularly in the decidualising endometrial stroma [27]. Throughout the menstrual cycle, uNK cells become the dominant immune cell population (>70%) to promote trophoblast invasion and spiral arteriole remodelling [28,29].

### 2.2. Endometrial Composition Changes during Pregnancy Establishment

The endometrial environment is fundamental for the establishment of pregnancy. After fertilisation and the journey down the oviduct to the uterine cavity, the blastocyst adheres to the endometrial luminal epithelium. This epithelium is crucial in mediating crosstalk between the implanting blastocyst and the endometrium [30,31]. Following implantation, the human embryo is completely enveloped within the endometrial lining by post-conception day 10 [32]. Extravillous trophoblastic cells (EVTs) rapidly proliferate and migrate into the decidua basalis. EVTs invade the underlying endometrial glands to anchor the conceptus to the uterine wall [31] and the spiral arterioles within the endometrium to remodel them into a low resistance, high flow system to allow for gas and nutrient exchange. The invasion and subsequent lining of the spiral arterioles by EVTs is crucial to the establishment of a well-functioning placenta. Paracrine signalling and histotroph secretions from the endometrial glands promote this process and provide nutrition to the conceptus until placentation is well established [33]. Whilst the placenta is critical to the maintenance of pregnancy, it is becoming clearer that the endometrial environment and maternal contribution have a huge influence on implantation and subsequent placentation to establish pregnancy.

## 3. The Origins of Adverse Pregnancy Outcomes

### 3.1. Parturition and Pregnancy Outcomes: Is Decidualisation the Determining Factor?

During the mid-secretory phase of each menstrual cycle, endometrial stromal cells commence a terminal morphological and functional differentiation termed decidualisation [34]. Elongated fibroblast-like cells transform into enlarged, round-shaped, highly secretory decidual cells, characterised by the release of insulin-like growth factor binding protein-1 (IGFBP-1) and prolactin (PRL) in preparation for pregnancy [35]. In response to elevated progesterone levels [36], decidualised stromal cells promote angiogenesis, activate matrix metalloproteinases (MMPs) and generate prostaglandins to facilitate trophoblast invasion and prevent immune rejection by recruiting uNK cells to the decidua [34].

Most non-menstruating eutherian mammals undergo decidualisation induced by the arrival of an implanting blastocyst; however, in humans, decidualisation occurs spontaneously [36,37]. The evolution of spontaneous decidualisation and menstruation is thought to coincide with invasive placentation, as decidual cells promote endometrial homeostasis during placentation [38,39]. Thus, in non-conception cycles, it is the terminally differentiated decidualised endometrium of the functionalis layer that is shed during menstruation in humans. The proliferative and differentiation capacity of decidual cells, which are a key determinant of menstrual health and pregnancy outcomes [14,40,41], can be assessed via endometrial and menstrual fluid samples in vitro [42].

There is abundant evidence that impaired decidualisation affects endometrial receptivity regarding early pregnancy loss and infertility [43], and this has also been proposed as a contributing factor to later adverse pregnancy outcomes [44]. Aberrations in the endometrial environment due to impaired decidualisation have been termed ‘decidualisation resistance’ [9] or maternal immune intolerance. Decidualisation resistance impacts trophoblast invasion, histotroph secretions and spiral artery remodelling, which can cause recurrent implantation failure, underlying infertility, or miscarriage [9,45,46]. Likely, decidual resistance is also involved in the pathogenesis of later pregnancy complications through several mechanisms [11,13,14,47,48], though the specific contribution of the endometrial environment to these adverse pregnancy outcomes remains largely unknown. Well-established hypotheses include decidual resistance impeding spiral arteriole remodelling, contributing to placental insufficiency in preeclampsia and FGR, and downstream effects on progesterone receptor expression leading to spontaneous preterm birth [11,14,48,49]. Most knowledge of endometrial associations with adverse pregnancy outcomes comes from circulating biomarkers present in peripheral blood [50,51,52,53] or retrospective analysis of placental tissue [54], and there is limited research using endometrial biospecimens themselves.

Many of the mediators involved in menstruation are also activated during labour, such as prostaglandins, pro-inflammatory cytokines and MMPs, which breakdown the endometrium for menstruation or degrade the extracellular matrix to weaken foetal membranes for rupture during labour [55,56,57,58,59]. While the exact mechanisms for initiating labour, both at term and preterm, are yet to be fully elucidated, it has been hypothesised that a ‘decidual clock’ is responsible for parturition, and as such, the decidua is vital in determining the onset of labour [60]. When decidual support is withdrawn, separation of the chorioamniotic membranes is initiated. Paracrine signalling by interleukins (IL) and prostaglandins between the endometrium and myometrium causes the switch between anti-inflammatory and pro-inflammatory states, which indicates the transition from a quiescent to a contractile phase. Cervical ripening in preparation for dilation is also mediated by changes in MMPs and inflammatory cytokines [61]. In the instance of spontaneous preterm birth, activation of inflammatory pathways likely signals a premature stimulation of the cascade of hormones responsible for parturition, though it remains unknown why this occurs (in cases that do not involve infection).

### 3.2. Evidence for the Endometrial Contribution to Adverse Pregnancy Outcomes

Later gestation pregnancy complications, including preeclampsia, FGR, spontaneous preterm birth and perinatal death, represent a continuum of adverse outcomes. These are often attributed to ‘placental insufficiency’ and share common pathways. Below, we provide detailed evidence of endometrial factors hypothesised to be involved in the development of adverse pregnancy outcomes. These factors are indicative of an altered endometrial environment and could be further explored by investigating menstrual health.

#### 3.2.1. Preeclampsia

Preeclampsia is a progressive hypertensive disorder that worsens as pregnancy continues [62] and is estimated to affect approximately 5% of all pregnancies [63]. Preeclampsia is largely considered to be a placental disorder, and as such, most preeclampsia research has been conducted on the placenta itself [63]. It is believed to stem from shallow trophoblast invasion and inadequate spiral arteriole remodelling, which subsequently leads to narrow maternal vessels and vascular resistance, eventually resulting in placental insufficiency. The contribution of the endometrium to this process, however, has not been fully explored, but there is evidence to suggest that decidualisation plays a crucial role.

Decidualisation assays on cultured human endometrial stromal cells derived from endometrial biopsies have shown reduced decidualisation marker expression (PRL and IGFBP1) and a failure to decidualise in vitro in women with a history of preeclampsia [11]. These findings were replicated in sections of decidua basalis and parietalis collected at the time of delivery from women with preeclampsia and showed a functional reduction in cytotrophoblast invasion [11]. Further transcriptional analyses of both endometrial stromal cells and the decidua basalis in cases of preeclampsia have demonstrated over a hundred differentially expressed genes, demonstrating impaired decidualisation and revealing the maternal contribution to preeclampsia [48,64]. Another study analysing the gene expression of chorionic villous samples showed that the genes dysregulated in preeclamptic cases were of decidual origin [48] and showed concordance with nine genes identified in the previous study [11]. Both studies also demonstrated differential gene expression of maternal immune cells, NK and T-cell receptors [11,48].

Several inflammatory cytokines, secreted by endometrial macrophages, have also been implicated in the pathogenesis of preeclampsia. The overexpression of circulating IL-6, IL-8, IL-1β and tumour necrosis factor-alpha (TNF-α) in cases of preeclampsia maintain a chronic pro-inflammatory state [51]. There is evidence that the source of excess circulating interleukins is the decidua, not the placenta, in preeclamptic cases compared to gestational age-matched controls [65]. Overexpression of these factors may act through varying mechanisms; for example, TNF-α impacts the ability of uNK cells to regulate the level of trophoblast migration and invasion [66]. Overexpressed TNF-α can also promote increased oestrogen biosynthesis in endometrial glandular epithelial cells. This is associated with the development of endometrial disorders such as endometriosis [67], which has an increased risk of preeclampsia [68].

This highlights the crucial importance of considering the endometrium in cases of preeclampsia. Even though the placenta retains some endometrial epithelial cells [69], very little research has been conducted on the pre-placental origins of preeclampsia. Further research into endometrial biomarkers for preeclampsia may provide novel insights into the pathogenesis of preeclampsia.

#### 3.2.2. Foetal Growth Restriction

FGR is defined as the inability of a foetus to reach its intrauterine growth and development potential due to placental compromise [70] and remains a leading cause of perinatal mortality worldwide. More than half of FGR cases remain idiopathic [71], as current clinical detection and diagnosis of FGR remains poor, with as many as four out of five growth-restricted babies remaining undetected in utero [72]. FGR is generally defined as a foetus weighing below the 10th percentile; however, not all foetuses that are small for gestational age have FGR. Incorrectly identified FGR can result in unnecessary harmful interventions, including iatrogenic preterm birth [73]. This can carry significant neonatal comorbidities such as cognitive deficits and cardiovascular disease later in life [74,75].

In the absence of genetic or structural defects in the foetus, placental insufficiency accounts for the majority of FGR cases [76]. A spectrum of placental pathologies contributes to uteroplacental insufficiency, ranging from impaired villous development to deficient vascular remodelling, resulting in inadequate nutrient transfer and foetal hypoxia [77]. There is emerging evidence that FGR and preeclampsia share common pathways, with a pro-inflammatory bias of increased levels of IL-6, IL-8 and TNF-α; however, FGR has been associated with decreased levels of the anti-inflammatory cytokine IL-10 in peripheral blood [53,78]. IL-10 suppresses natural killer-like cells at the uteroplacental interface, and cases of FGR demonstrate a reduced proportion of uNK cells in the decidua basalis compared to controls [79]. Interestingly, this association was significant with FGR, regardless of preeclampsia status; however, the reduction was not significant in isolated cases of preeclampsia without FGR [79]. The balanced composition of decidual leukocytes is crucial for the maintenance of pregnancy, and dysregulation of the decidua may be a prominent early event of pregnancy that affects the regulation of inflammation and spiral artery remodelling. It is unknown what causes this initial dysregulation and subsequent inflammatory responses in the endometrial environment and why this manifests differently in some cases of preeclampsia and FGR.

#### 3.2.3. Spontaneous Preterm Birth

Spontaneous preterm birth remains one of the leading causes of perinatal mortality in high-income countries [5,80]. Some of the top risk factors for spontaneous preterm birth include prior preterm birth [81] and preeclampsia [82]. As many as two-thirds of preterm births may occur in the absence of any evident risk factors [83], and the underlying mechanisms leading to spontaneous preterm birth are largely unknown.

It has been previously proposed that the four following interrelated pathogenic mechanisms cause spontaneous preterm birth [84]:

1. Activation of the maternal or foetal hypothalamic-pituitary-adrenal (HPA) axis;
2. Decidual-chorioamniotic or systemic inflammation;
3. Decidual haemorrhage (abruption);
4. Pathological distention of the uterus [84].

Similar to the spectrum of adverse pregnancy outcomes, it is likely that the origins and severity of spontaneous preterm births also lay on a continuum dependent on the initial endometrial environment and may stem from underlying endometrial abnormalities. This hypothesis is supported in a review by Ng et al. [9], which postulates that these processes are interrelated, as evidenced by overlapping biomarkers and risk factors for other adverse pregnancy outcomes with preterm birth (Figure 1).

Many of the molecular mechanisms implicated in the pathogenesis of preeclampsia and FGR discussed above are also dysregulated in cases of spontaneous preterm birth, and identifying biomarkers for spontaneous preterm birth remains challenging. A cross-sectional study of urine and plasma collected from women who experienced spontaneous preterm birth demonstrated that TNF-α, IL-6, IL-10 and IL-1β were all positively associated with an increased risk for spontaneous preterm birth; however, only IL-10 was found to be statistically significant [50]. IL-10 is specifically elevated among cases of preterm birth with aberrant placentation [85,86]. Whilst of interest, it is unlikely that plasma and urine samples are exclusively representative of the endometrial environment, and as highlighted above, the source of these circulating factors and whether differential expression of these mediators is implicated in endometrial cells requires further investigation.

This could be achieved with a focus on studying menstrual fluid composition in women with pregnancies previously impacted by spontaneous preterm labour. For instance, the expression of MMP-1 and MMP-9 is higher in the placental tissue of preterm [54] compared with term births. The very nature of placental investigations, however, is retrospective, and it can be difficult to separate the foetal and maternal contributions. These factors can be detected in endometrial tissue derived from menstrual fluid, and MMP-1 has been shown to be significantly upregulated in the menstrual fluid of infertility cases [87]. Further research comparing placental tissue with endometrial tissues would determine whether these factors can be used as biomarkers in preconception care to predict an increased risk for spontaneous preterm birth.

#### 3.2.4. Perinatal Death

Perinatal death can occur as a result of the aforementioned pregnancy complications. Regardless of the gestation or cause, perinatal death is a traumatic experience that has a profound impact on all of those affected. Due to the logistical considerations of obtaining endometrial samples from women impacted by pregnancy and infant loss, there has been very little research into the exact endometrial contribution. However, both uterine maturity [88,89,90,91] and decidualisation capacity [92] have been implicated in pregnancy loss, highlighting the importance of exploring this concept further.

As progesterone is essential to maintaining the menstrual cycle, there is a theory that cyclic menstruation ‘preconditions’ the uterus for pregnancy by protecting the uterus from inflammatory and oxidative stress associated with placentation [88]. This may explain why adolescent pregnancies (<20 years old) have a higher risk of perinatal death and adverse pregnancy outcomes [89,90]. This has often been attributed to socioeconomic disadvantage and insufficient reproductive education associated with teenage pregnancy [91]. Uterine immaturity due to progesterone resistance, however, has also been indicated as a potential underlying factor that impacts endocrine pathways and decidualisation capacity [92]. This is evident when we consider that neonates have inactive endometrium, and females transition to a state of cyclic ovarian oestrogen and progesterone production, followed by progesterone responsiveness during puberty. This then triggers the onset of regular menstruation and decidual gene expression [92]. Inadequate vascular remodelling in adolescent pregnancies may be a result of incomplete cyclic programming of uNK cells. The higher rate of stillbirth among adolescent pregnancies may be an indication of insufficient menstrual cycles to precondition the uterus for adaptation to pregnancy [88]. This highlights the significance of the functional decidua for both a healthy menstrual cycle and pregnancy.

Conversely, advanced maternal age (>35 years old) is also a risk factor for perinatal deaths [93]. This is often attributed to declining oocyte quality and increased rates of embryonic chromosomal abnormalities. Emerging evidence from preclinical models, however, indicates that most pregnancies with later adverse outcomes occur in the absence of a chromosomal abnormality, indicating a role for the uterine environment [94]. Age-related inflammation occurs in the endometrium [95], and it has been demonstrated in mice models that advanced maternal age interferes with the progesterone response of stromal cells, resulting in a reduced capacity to decidualise [94]. This has downstream implications for placental establishment and function, which has been shown to gradually decrease in a maternal age-dependent manner via reduced concentration of pro-inflammatory cytokines IL-1β and TNF-α [96]. Placentas from advanced-aged mothers also weigh significantly more even though there is an increased incidence of FGR [96,97], which may be indicative of a response to compensate for poor placental function. This evidence emphasises the complexity of elucidating the molecular mechanisms underpinning pregnancy complications but reiterates that a healthy endometrium is key to maintaining pregnancy.

## 4. Is Menstrual Assessment the New Frontier in Understanding the Endometrial Contribution to Adverse Pregnancy Outcomes?

The complexities of menstruation, conception, decidualisation, implantation, placentation and parturition are inevitably difficult to replicate and study in vivo in humans. As detailed in the previous sections, understanding of these processes and the errors that occur in these pathways have largely come from animal models, placental examinations and in vitro studies using tissue from endometrial biopsies. Given the copious molecular mechanisms that are spatiotemporally regulated during implantation and placentation, retrospective placental examination is inadequate to gain the critical insights required to understand the aetiology of these adverse pregnancy outcomes. Many of these approaches are also time-consuming and invasive, and sample sizes for studies are often small due to difficulty recruiting relevant populations to prospective clinical studies. The potential to uncover the endometrium’s contribution to adverse pregnancy outcomes via menstrual history taking, clinical assessments and biological evaluation of menstrual fluid can overcome these challenges, paving the way to drive this important research field forward.

### 4.1. The Potential for Investigating Menstrual Characteristics Associated with Adverse Pregnancy Outcomes

Menstrual characteristics, including age at menarche, cycle length, period regularity, length and heaviness, period pain and associated menstrual symptoms, are all indications of menstrual health.

There is some evidence for the association between certain menstrual characteristics and adverse pregnancy outcomes. Early studies in the 1980s indicated that there may be a higher risk of miscarriage [98], spontaneous abortion [99] and ectopic pregnancy [100] in women who experience early menarche, traditionally defined as the onset of menstruation before 12 years old. Early menarche has since been associated with a higher risk of preeclampsia [101,102,103,104], preterm birth [16] and the likelihood of a low birth weight baby [102]. Interestingly, self-reported heavy or irregular periods prior to a second or subsequent birth in multiparous women were also associated with an increased risk of preterm birth in the subsequent pregnancy, demonstrating that new menstrual symptoms may arise after pregnancy [105]. There is also evidence of an increased risk for preterm birth when there is prolonged menstruation before conception [106], likely due to an extended proliferative phase. This implies that pregnancy, where implantation occurs outside of the critical fertile window, may still progress but have consequences that manifest later in pregnancy. Despite this, most antenatal assessments only obtain clinical information about the date of the last menstrual period to determine an estimated birth due date, with no consideration of previous menstrual health. This could be a critically missed opportunity to gather more information about the endometrial environment.

Abnormal menstruation and adverse pregnancy outcomes may have common causes that effect the endometrium. For example, both increased and decreased body mass index (BMI) is known to impact the menstrual cycle and is often attributed to hormonal factors. Increased BMI is associated with heavy menstrual bleeding and increased risk of premenstrual disorders [107], whereas decreased BMI is associated with irregular menstruation or amenorrhea (the absence of menstruation), and both report painful periods more frequently than those with an average BMI [108]. Maternal pre-pregnancy BMI also increases the risk of preeclampsia [101,109]. This is thought to be caused by influencing the length of the menstrual cycle, and thus, differential expression of endometrial receptors throughout the menstrual cycle may result in inadequate trophoblast invasion and subsequent placental insufficiency [101,109]. Rather than implicating a direct cause-and-effect model, we emphasise that the association between menstrual characteristics and pregnancy outcomes can be used as a proxy measure of endometrial health to warrant further investigation.

### 4.2. The Potential for Analysing Menstrual Fluid Regarding Adverse Pregnancy Outcomes

Menstrual fluid can be collected non-invasively and used to assess the composition of the endometrial environment (reviewed in [110]). Menstrual fluid demonstrates a reproducible profile with minimal variation between menstrual cycles, indicating that it is an appropriate biospecimen representative of an individual’s endometrial environment [17]. Whilst menstrual fluid has recently been adopted to study endometriosis [18,19,111,112,113,114,115,116,117,118] and unexplained infertility [45,98,99,119], there is no evidence of its use in investigating adverse pregnancy outcomes. We are currently conducting a case–control study, where participants who have experienced adverse pregnancy outcomes in the second and third trimesters donate menstrual fluid samples for molecular analyses [100]. In this study, women who have experienced a preterm stillbirth or livebirth (20–36 + 6 weeks’ gestation), FGR (birth weight <3rd centile by population charts [101]) or second-trimester miscarriage in the past 3 years (cases) and those who have not (healthy term birth matched for maternal age, BMI and gravidity) (controls) are being recruited [100]. Differences in cellular and immune cell composition, secreted proteins and menstrual cycle characteristics are being compared [100]. The potential for this research is just emerging, and modern techniques, such as endometrial organoids derived from menstrual fluid, provide a particularly exciting opportunity to understand the endometrial contribution to adverse pregnancy outcomes better than ever through in vitro experimentation [19,102]. Placental organoids can also be co-cultured with paired endometrial samples to elucidate these mechanisms further.

### 4.3. Limitations and Opportunities for Assessing the Associations between Menstruation and Pregnancy Outcomes

We acknowledge the limitations that are inherent in a narrative review; however, this methodology was appropriate given the sparse current evidence connecting menstruation and pregnancy outcomes. The aim of this review was to collate evidence for the endometrial contribution to adverse pregnancy outcomes, understand the landscape of menstrual associations with pregnancy outcomes, and provide a novel hypothesis as further rationale to address this as a research question.

Menstruation is a unique biological process, observed in only 1.6% of all eutherian species [103], making it difficult to study in preclinical settings. Even though approximately half of the human population menstruate throughout their reproductive life, menstruation remains a taboo topic in public discourse. Menstrual fluid has been referred to as ‘one of nature’s most stigmatized fluids’ [104]. For example, it was not until 2023 that real blood was used to measure the absorbability of menstrual sanitary products to assess heavy menstrual bleeding [109]. Menstrual blood, or menstrual fluid, itself has still never been utilised for this purpose.

There are many advantages to using menstrual fluid samples for research, including ease and non-invasiveness of collection and the fact that it is readily obtainable from reproductive-aged women. Up to 80% of women are willing to donate menstrual fluid for medical research [105,106], indicating that it is a feasible collection method. When surveyed about their perspectives on donating menstrual fluid, some women had hygiene concerns, but most responded positively. Reasons to donate menstrual fluid included current use of a menstrual cup, which is environmentally friendly, and it being an empowering, easy and “excellent use of a waste product” [105]. If advertised correctly and sensitively, such research may also give women a sense of purpose for their menses, with one woman stating, “If I felt I could help in any way to improve the lives of others in such a simple way it can only be a good thing” [105].

Sampling shed endometrium using menstrual fluid mitigates the risk of cycle bias, which is often not accounted for with endometrial biopsies. Due to the cyclical nature of menstruation, though, menstrual fluid may not accurately reflect the endometrium during earlier phases of the menstrual cycle and should be considered in the context of the late-secretory phase. For example, drawing inferences from immune cell proportions at one time-point may not be indicative of an abnormality throughout the menstrual cycle or pregnancy. Findings may still require comparison with endometrial tissue during other menstrual cycle phases or gestational-age-matched placental tissue samples to investigate if this phenotype persists.

Gathering a menstrual history over time and in the preconception phase provides valuable insight into endometrial health and potential future pregnancies. While concerns of recall bias have been expressed when taking menstrual histories, most women recall their menstrual history accurately [120], and for those who do not, the ever-changing nature of menstruation throughout reproductive life may be the reason. With the emergence of modern period-tracking apps and enhanced reproductive education, menstrual tracking is simple, valid and easier than ever.

Pregnancy loss and infertility also remain highly stigmatised topics [121,122]. Collecting menstrual fluid samples from women who have experienced traumatic pregnancy outcomes may make participating in such studies emotionally challenging and should be treated sensitively [123]. There is an underwhelming amount of research connecting these two areas, and the associated taboos have engrained social and systemic barriers to conducting such research. For such research to prevail, successful participant recruitment relies on addressing the taboos that underlie these barriers.

As highlighted in Figure 1, numerous other factors contribute to establishing and progressing pregnancy. Various confounders include nutrition, the environment, maternal and paternal conditions and anomalies of the foetus itself. We also acknowledge that with advancements in assisted reproductive technologies, menstruation is not necessitated to achieve pregnancy. Women who are postmenopausal or do not menstruate following a uterine transplant can now become pregnant. We emphasise here, though, that the endometrial contribution to pregnancy success has been underestimated and under-researched to date and that a better understanding of menstruation can aid further progress.

### 4.4. Elucidating the Endometrial Environment via Menstrual Assessment: What’s Next?

For this research to progress, several things must be addressed. Firstly, standardised definitions for what constitutes ‘abnormal’ menstruation should be updated and implemented. A classification system for symptoms and causes of abnormal uterine bleeding was established in 2011 and revised in 2018 [124]; however, it is still not routinely used in clinical practice. It is essential that we can delineate between idiopathic abnormal menstruation and endometrial pathologies. Abnormal menstruation may be more prevalent than previously realised, and updated criteria will aid pathways for those that warrant further investigation regarding potential pregnancy outcomes. Large datasets from period-tracking apps may challenge current definitions of abnormal menstruation and reveal which menstrual characteristics are most helpful for assessing pregnancy risk.

If the menstrual fluid is to be pursued as a legitimate sample for biochemical analyses, further characterisation of menstrual fluid composition and normal parameters should also be determined. For this to occur, a standardised protocol for the optimal collection, processing, storage and experimentation of menstrual fluid must be established. In our recent review of menstrual fluid, we detail how this can be achieved [110].

Furthermore, during this review, we identified crucial knowledge gaps regarding the endometrial contribution to adverse pregnancy outcomes. A recent systematic review revealed that of 74 endometrial transcriptome studies, all were conducted regarding menstrual cycle differences, endometrial and fertility-related pathologies and response to hormone treatment [125]. None of these studies investigated adverse pregnancy outcomes. We emphasise the need for further scientific discovery, particularly investigating genetic and epigenetic differences in endometrial tissue related to adverse pregnancy outcomes, to explicate upstream mechanisms of observations at the functional level.

## 5. Conclusions

Menstrual health and women’s health are finally being drawn into focus, and now is the time for advanced investigations into the links between menstruation, the endometrium and pregnancy [126]. As detailed here, there is increasing data regarding the endometrial contribution to adverse pregnancy outcomes; however, most evidence is derived from invasive endometrial biopsies, peripheral blood biomarkers, preclinical models, or retrospective placental examinations. We and others hypothesise that understanding menstrual history and menstrual fluid collection is a non-invasive and novel approach for better understanding the endometrial environment regarding adverse pregnancy outcomes [9,38,103,127]; however, this call to action has not yet been answered. Exploring this avenue of research will lead to identifying completely new and undiscovered drivers of poor placentation, unravelling the potential for preconception biomarkers and therapies to mitigate some of the greatest causes of death, disability and heartbreak that is seen in obstetrics, including preeclampsia, FGR, preterm birth and perinatal death.
